# Impact of an interdisciplinary digital consultation platform on general practitioner referrals for musculoskeletal symptoms: a stepped wedge cluster randomized trial

**DOI:** 10.1093/fampra/cmaf071

**Published:** 2025-09-19

**Authors:** Sanne M Sanavro, Henk van der Worp, Henk Schers, Joke Stoffelen, Clarinda van den Bosch, Joris van Dijk, Petra Buist, Michiel R de Boer, Guus J M Janus, Marco H Blanker

**Affiliations:** Department of Primary and Long-Term Care, University of Groningen, University Medical Center Groningen, 9713 GZ Groningen, The Netherlands; Department of Primary and Long-Term Care, University of Groningen, University Medical Center Groningen, 9713 GZ Groningen, The Netherlands; Department of Primary and Community Care, Radboud University Nijmegen Medical Centre, 6525 EZ Nijmegen, The Netherlands; Zorgbelang Inclusief, 6811 JH Arnhem, The Netherlands; Isala Clinics, 8025 AB Zwolle, The Netherlands; Isala Clinics, 8025 AB Zwolle, The Netherlands; Department of Primary and Long-Term Care, University of Groningen, University Medical Center Groningen, 9713 GZ Groningen, The Netherlands; Department of Primary and Long-Term Care, University of Groningen, University Medical Center Groningen, 9713 GZ Groningen, The Netherlands; Isala Clinics, 8025 AB Zwolle, The Netherlands; Department of Primary and Long-Term Care, University of Groningen, University Medical Center Groningen, 9713 GZ Groningen, The Netherlands

**Keywords:** digital health, telemedicine, randomized controlled trial, referral and consultation, orthopaedics, primary health care

## Abstract

**Background:**

The aim of the study was to assess the effect of an interdisciplinary, digital consultation platform on the proportion of appropriate referrals from general practitioners (GPs) to an orthopaedic outpatient hospital.

**Methods:**

We performed a stepped wedge, cluster, randomized controlled trial. Sixty GP practices in the catchment area of a large teaching hospital in the Netherlands were randomized. Groups of GP practices entered the trial in four steps at 13-week intervals, at which point they received access to the Prisma platform. The platform allowed them to post questions about anonymized cases to a multidisciplinary group of specialists. During the control condition, GPs did not receive platform access. In both conditions, GPs provided care as usual. The proportion of appropriate referrals, defined as referrals for which a patient had either (i) more than one consultation with an orthopaedic surgeon or (ii) one consultation with additional diagnostics or interventions, was the primary outcome.

**Results:**

Participating GPs referred 4928 patients to hospital. Intention-to-treat analysis showed a 4.4% estimated increase in the proportion of appropriate referrals among GP practices randomized to have access to the platform compared to the control group, with an odds ratio (OR) of 1.22 [95% confidence interval (CI), 1.01–1.46; *P* = 0.037]. Per-protocol analysis showed a smaller, but non-significant, 2.2% difference between interventions, with an OR of 1.11 (95% CI, of 0.96%–1.28%; *P* = 0.178).

**Conclusions:**

We observed a modest increase in appropriate referrals for orthopaedic review among GP practices randomized to the platform. On a larger scale, this could contribute to more sustainable access to specialist care.

Key messages

- The platform had a modest positive effect on the appropriateness of referrals.
- The platform could contribute to more sustainable access to specialist care.
- We are reluctant to draw firm conclusions due to the complexity of the intervention

## Background

Dutch general practitioners (GPs) are able to manage musculoskeletal conditions conservatively in symptomatic patients and typically act as gatekeepers for referral to hospital specialists [1, 2]. Nevertheless, a significant number of patients are still referred for orthopaedic surgery consultations, with knee, hip, and shoulder conditions accounting for 29%, 5.5%, and 10%, respectively [3]. Many of these referred cases only receive generic treatment in secondary care, such as analgesia or physiotherapy referral which are also available in primary care. This suggests that referral was potentially avoidable and raises the question whether prior consultation between a GP and a specialist could have prevented the secondary care appointment.

How GPs manage diagnostic and treatment uncertainty plays an important role in decisions about patient referral [4]. Having an efficient and accessible way of communicating with orthopaedic surgeons when there is doubt about the appropriateness of a referral could, therefore, help GPs make more informed decisions [5]. Given that synchronizing telephone availability between specialists and GPs can be challenging, the use of digital platforms has become an acceptable method of interdisciplinary consultation. Unidirectional digital consultations (one question, one answer) can prevent “inappropriate” patient referrals to specialists for a variety of conditions and are appreciated for their educational value [6–10]. However, this consultation model may not suit all queries, such as when the question is more general, or the case is complex.

Most digital consultations merely reflect one opinion, are unidirectional, and do not stimulate the teamwork that is often necessary for optimal patient-centred healthcare. In the long term, it is also inefficient for advice to be given from one specialist to one GP, because valuable knowledge is not stored for later use or learning by others [7, 11]. Collaboration within a network of physicians could offer a solution to the shortcomings of one-on-one consultations. An example is the Prisma consultation platform in the Netherlands, which facilitates asynchronous consultations with digital storage in a searchable format. An overview of the platform's activity demonstrated that it provides an effective route for rapid interdisciplinary collaboration, allowing both knowledge exchange and storage [12]. Potentially, such a platform could affect when GPs make referrals to hospital. To date, however, no comparative research or initiative has been performed to support this assumption.

We conducted a trial to analyse how introducing and implementing the platform in GP practices affected the proportion of appropriate referrals to specialist services in hospital. Specifically, the use of the platform was expected to reduce the proportion of inappropriate referrals to hospital specialists for knee, hip, and shoulder complaints.

## Methods

### Study design

This stepped wedge, cluster, randomized controlled trial (RCT) included GPs in the east of the Netherlands from September 2021 to December 2022. The study protocol, selection considerations, and adjustments have been published previously [13, 14]. The stepped wedge design allowed for the evaluation of this new healthcare intervention for implementation at the organizational level given the expectation of logistical challenges related to onboarding an entire region simultaneously and in ensuring timely responses from specialists to the resulting surge in posted cases [15].

GP participants were enrolled from among those working in the catchment area of a single large teaching hospital (Isala) in Zwolle, the Netherlands. The region has around 350,000 inhabitants. This region was chosen for two reasons. First, it contained a negligible number of GPs already using the intervention, preventing contamination in the control group. Second, the region was served by only one hospital to which GPs could refer most patients for specialist consultation or additional testing (i.e. laboratory tests and radiology).

The Medical Ethical Review board of the University Medical Centre Groningen waived the requirement for ethical approval (METc-number: 2021/288). The trial was registered with the Dutch Trial Register (NL-OMON23624) and is reported according to the extended CONSORT checklist for reporting stepped wedge trials [16].

### Sample size calculation

Based on an average of four GPs per practice in the study region, we expected approximately 56 referrals annually per practice [1–3]. Projecting a 26% reduction in referrals with the intervention based on the results of a pilot study, an estimated 348 patients or referrals needed to be included for comparison to achieve a significance level (alpha) of 0.05 and a power of 90%. The intra-cluster correlation coefficient was estimated to be 0.2, assuming similar referral patterns within groups. A design effect due to cluster randomization was calculated at 3.6, and given an assumed cluster autocorrelation of 0.9, the design effect due to repeated assessments was calculated at 0.1913. Therefore, we needed to include 18 practices (3.6 × 0.19 × [348/14]) with 1008 patients for potential referral. Assuming only 60% of the practices invited took part, we would need to include 40 practices and 2240 potential referrals to detect a 16% reduction (instead of the expected 26%).

### Randomization, allocation concealment, and blinding

Randomization at the GP practice level created practice clusters of GPs implementing the intervention. A regional support group assigned each practice a unique study code. The researchers used these data to create a blinded randomization list stratified by practice location (city vs rural) and group size (small, 1–2 GPs; large, ≥3 GPs), and created four sequences that each introduced between 14 and 16 practice clusters to the intervention group at four different steps. The first group was invited to join the platform on 15 September 2021. Thereafter, a new group was invited to join the intervention group at 13-week intervals. After inclusion, practice clusters remained in the intervention group until trial completion; locum GPs working in different clusters were asked to use the platform only in the clusters where the intervention had been rolled out.

The regional support group sent letters to each GP in each practice with instructions about joining the platform and the onboarding procedures. Researchers, GP participants, and patients were blinded to the random sequence allocation, but the study's design precluded blinding the researchers, GP participants, and patients during the intervention.

At the end of the trial period, but before data extraction, we added an unplanned extra observation period of 13 weeks [13]. This alteration to the original study design was necessary because of slow uptake of the intervention by GP practices. For each referred patient, a follow-up interval of 120 days after the patient's first consultation with an orthopaedic surgeon was considered appropriate to provide all the necessary data for the primary outcome. This data also included information on whether the patient was scheduled for surgery at 120 days.

### Intervention: access to the platform

The study intervention provided access to the full Prisma platform. This is a digital, asynchronous, interdisciplinary consultation platform for use by physicians. It allows GPs to post non-urgent anonymous questions to specialists who are grouped into teams by specialty or topic. All physicians can post, read posts, offer advice, and discuss cases, with all communication stored in a searchable database named the “knowledge bank” [14].

Each GP received a personal account with instructions on how to use the platform and reminders sent to activate their account. However, GPs could choose whether to use the platform and, if so, how intensively and for what topics or specialty. For the control condition, GPs had no access to the platform. For both the control and intervention groups, GPs provided care as usual at their discretion. The ability to inform decisions based on advice given on the platform differentiated the intervention condition from the control condition. There were no costs for using the platform.

### Patient and public involvement

A patient representative (Zorgbelang Inclusief) was involved in the conception and design of the study and the interpretation of the results.

### Study outcomes

The primary outcome was the proportion of appropriate referrals from GPs to the department of orthopaedic surgery in the Isala Movement Centre during the trial period. This study focused on adult patients with knee, hip, or shoulder symptoms and excluded patients with acute trauma. An appropriate referral was defined as a first outpatient visit with an orthopaedic surgeon (including surgeons in training, physician's assistants, and nurse practitioners) with at least one of the following additional criteria: follow-up (telephone) consultation within 120 days; additional radiology, except for same day X-ray; or an intervention planned within 120 days. Data for the primary outcome were obtained from the hospital using the diagnosis treatment combination system. This allowed the creation of a dataset that contained all recorded activities per referring GP practice for each referred patient, along with exact referral dates. We also calculated the surgical yield, defined as the percentage of referrals leading to scheduling of a surgical intervention, for both conditions.

The study window included patients referred from April 2021 to December 2022.

### Statistical analysis

We performed intention-to-treat (ITT), per-protocol (PP), and post hoc analyses for the primary outcome [13].

All participating GPs were included by randomization group for the ITT analysis; however, for the PP analysis, practices were only included in the intervention condition from the moment a GP (a practice partner or locum) activated their account to view at least one case. Where a locum triggered inclusion for the PP analysis, we only included a practice in the intervention condition if the locum had worked in the practice for at least 13 weeks and at least 2 days per week. GP practices not meeting these conditions remained in the control condition for the PP analysis.

For the ITT analysis, we used the full exposure time based on the randomization group. For the PP analysis, we used the actual exposure time of the practice using log data from the platform.

We describe GP practice and patient characteristics. For the GP practices, we describe the median number (range) of GPs per practice and the characteristics of GPs within the practice [i.e. GP partners (owners), locum GPs, employed (salaried) GPs, and GPs in training]. We made a distinction between GP partners and employed GPs because in the Netherlands, GP practices are privately owned, and partners—being co-owners—typically have more influence on practice policies, including the use of consultation platforms, than employed GPs. Partners usually also spent more time on managing tasks whereas employed GPs are mainly involved in patient care. We described the number of GP practices per sequence in the intervention for both the ITT and PP analyses. For patient characteristics, we describe the total number of referred patients and appropriate referrals as percentages for the intervention and control conditions and for each step and cluster.

We used logistic mixed models to estimate the effect of using the intervention compared with usual care on the proportion of appropriate referrals, with referral appropriateness (yes/no) set as the dependent variable and condition (intervention versus control) set as the fixed effect of interest. The models included random intercepts at the GP practice level adjusted for time as a fixed effect and using dummy variables for differences between steps. We also included the covariates practice size (small/large) and location (city/rural) as fixed effects. Odds ratios (ORs) and 95% confidence intervals (95% CIs) are reported.

The platform's unique design allowed participants to search and re-use all multidisciplinary case-based advice held in the database. To explore the potential learning effect from the intervention, we added a post hoc analysis of the effect of intervention exposure time on the primary outcome before analysing the data. This involved a similar logistic mixed model analysis to the primary analysis, but with exposure to intervention (duration) as the fixed effect of interest, using dummy variables for the different exposure times in the ITT analysis and exposure time as a continuous variable in the PP analysis. Other fixed and random effects in this model did not differ from the primary analysis.

All analyses were performed using STATA, version 18.

## Results

**Figure 1.**
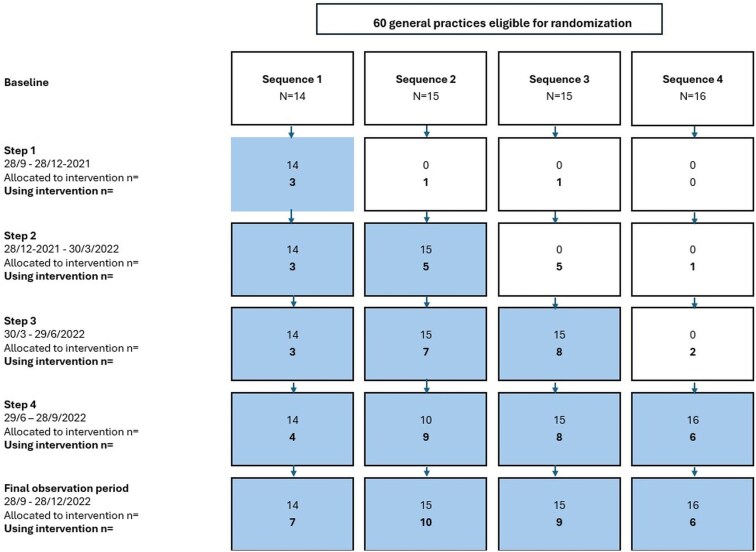
Flow chart of GP practice allocation and adherence for each cluster and step.

We randomized 60 practice clusters for analysis, of which 32 contributed to the PP analysis (Fig. 1). During the trial, six practice owners stopped providing patient care due to illness or retirement, but care of their patients was taken over by other GPs from the same practice. In total, 146 GP partners, 75 locum GPs, 6 employed GPs, and 13 GPs in training were included (Table 1). Because locums were able to work at more than one practice, we counted each locum workplace separately. Of the 262 GPs a 106 started using the intervention. The median uptake time from randomization to platform access was 22 days (range, 0 to 388 days) for onboarded practices.

**Table 1.** GP practice characteristics, percentage of GPs, and location by cluster.

	Cluster 1	Cluster 2	Cluster 3	Cluster 4	Total
Total number of GPs^a^	59	70	65	68	262
Practice partner (%)	31 (52.5)	46 (65.7)	32 (49.2)	37 (54.4)	146 (55.7)
Locum (%)a	23 (39.0)	22 (31.4)	28 (43.1)	24 (35.3)	97 (37.0)
Employed GP (%)	3 (5.1)	0	2 (3.1)	1 (1.5)	6 (2.3)
GPs in training (%)	2 (3.4)	2 (2.9)	3 (4.6)	6 (8.8)	13 (5.0)
Median GP/practice (range)	3.5 (2–9)	4.0 (2–9)	4.0 (1–10)	3.5 (1–13)	4.0 (1–13)
Practices	14	15	15	16	60
Rural practices	9	9	9	9	36
Urban practices	5	6	6	7	24

GP, general practitioners.

^a^Locums working in more than one practice were counted more than once.

GPs referred 4928 patients during the trial period, of which 2445 (49.6%), 1039 (21.1%), and 940 (19.1%) were for knee, hip, and shoulder diagnoses, respectively; 504 (10.2%) were for the diagnosis of other musculoskeletal conditions. Referred patients in the intervention and control conditions did not differ by age or gender (Supplementary S1).

The percentages of appropriate referrals per step and cluster are shown in Table 2. In the ITT analysis, 67.3% in the intervention condition and 62.9% in the control condition made appropriate referrals, showing a positive effect of the intervention on the proportion of appropriate referrals, with an estimated difference of 4.4% (95% CI, 0.03%–8.5%) and an OR of 1.22 (95% CI, 1.01–1.46; *P* = 0.037). In the PP analysis, 67.0% in the intervention condition and 64.8% in the control condition made appropriate referrals, showing an estimated difference of 2.2% (95% CI, −1.01% to 5.49%) and an OR of 1.11 (95% CI, of 0.96%–1.28%; *P* = 0.178).

**Table 2.** Observed appropriate and total referrals by step and cluster.

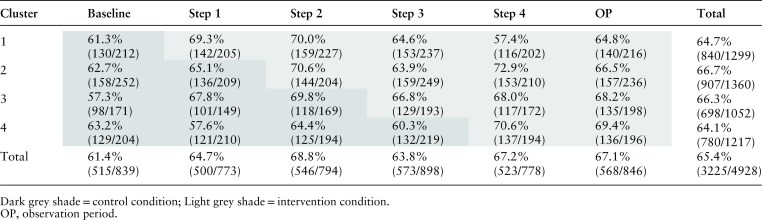

The post hoc ITT analysis of exposure time revealed the intervention had consistent effects until 39 weeks of exposure, after which effects were attenuated (Table 3). We observed appropriate referrals in 69.1% (13 weeks), 67.5% (26 weeks), and 68.1% (39 weeks) of cases, with ORs of 1.32 (95% CI, 1.11%–1.58%; *P* = 0.002), 1.23 (95% CI, 1.03–1.46%; *P* = 0.02), and 1.26 (95%CI, 1.04%–1.52%; *P* = 0.02), respectively, compared with no exposure.

**Table 3.** Impact of the Prisma platform on referral appropriateness in intention-to-treat, per-protocol, and post hoc analyses.

	OR	95% CI	*P*-value
ITT	1.22	1.01–1.46	0.04
PP	1.11	0.96–1.28	0.18
Post hoc			
ITT exposure time			
13 weeks	1.32	1.11–1.58	0.002
26 weeks	1.23	1.03–1.46	0.02
39 weeks	1.26	1.04–1.52	0.02
52 weeks	0.97	0.79–1.21	0.80
65 weeks	1.13	0.84–1.52	0.42
PP exposure time^a^	1.01	0.96–1.06	0.70

^a^Per 13 weeks additional intervention exposure.

CI, confidence interval; ITT, intention-to-treat; OR, odds ratio; PP, per-protocol.

Note: Covariates included in the model as fixed effects are time, practice size, and practice location.

In the PP analysis of exposure to the intervention for 13 additional weeks, we found no relationship to the proportion of appropriate referrals (OR, 1.01; 95%CI, 0.96–1.06; *P* = 0.70) (Table 3).

Surgical yield was 10.5% (209/1989) in the control condition and 13.1% (384/2939) in the intervention condition.

## Discussion

This stepped wedge RCT revealed a modest positive effect of the Prisma platform on appropriate referrals to orthopaedic surgery, showing a 4.4% increase. However, we expected a larger effect based on data from the pilot study we used for sample size calculation. GPs in the present study had much higher levels of appropriate referral at baseline compared with the pilot study, which probably led to a decrease in the potential for improvement [14]. Still, the total number of appropriate referrals in the region increased from 62.9% to 67.3%, which could have a significant impact on patient care if implemented on a larger scale. We also expected to find an accumulating effect with duration of exposure to the intervention because of its educational potential [6, 7, 17], but neither the ITT nor the PP analyses supported this effect. Overall, we remain hesitant about drawing strong conclusions about the causal relation between platform use and referral behaviour or educational potential because of the complexity and dynamics of platform use by GPs within participating practices.

To our knowledge, this is the first RCT to have observed a positive effect of an online platform on appropriate referrals (in this case, for orthopaedic surgery). A comparable, large, parallel arm RCT of 113 family physicians who completed 44 066 referrals to medical specialties in Canada found a 6% difference (relative risk, 0.93; 95% CI, 0.85–1.03). Although this was in the same range as our outcome, they measured the total number of referrals instead of the percentage of appropriate referrals [18]. Furthermore, most other studies have only examined one-way teleconsultations, making it difficult to perform direct comparison with our platform. Studies employing a variety of research methods have reported promising effects with the percentages of avoided referrals varying widely from 22% to 68% [19–22]. Two cross-sectional studies of orthopaedic surgery in Canada reported that referrals were avoided in approximately two-thirds of adults and children, and that GPs modified their referral behaviours after almost half of the teleconsultations. Contrasting with our study, these analysed teleconsultation data instead of hospital registration data, meaning that much larger effects are to be expected [21, 23].

This study has several strengths. First, we executed an ITT intervention in GP practices in a relatively “intervention naïve” study region, thereby creating a proper control condition. Although we learned that 66 of the 262 GPs had already registered on the platform before trial entry, exploring the activation log data indicated that only four had actively used the platform in the months before the trial started; given the small group size, we do not expect a large impact on the results. Second, we engaged regional partners to limit the impact of external factors during the trial period. These partners actively paused the introduction of new local guidelines and procedures in general practice and orthopaedic surgery, including any related educational activities for GPs. Third, we used real-world hospital data to measure our outcomes. To date, assessments of referral appropriateness have been more subjective, including triaging referral letters compared to guidelines, scoring e-consultations, and using post-consultation surveys of hospital specialists [19, 24, 25]. We acknowledge that our criterion for referral appropriateness is only a proxy measure that is not based on externally validated definitions. However, we know of no such definition in the literature, and argue that our criterion provided an objective measure of actual patient care that contrasts with the more subjective criteria used previously. We pre-defined our criteria on local workflow from expert opinion within the research team, using the proxy criteria reported for rheumatology consultations [26].

A potential limitation of the study design is our inability to link platform consultations to GP referrals for each patient. Having this option would have provided more information on the considerations made before referral, and it may have supported a more detailed interpretation of the found effect. However, even with this option, we would be lacking the potential circumstantial and indirect effect of our intervention, in which real-world information and knowledge could be re-used and shared among GPs. To reduce contamination, we consider it justifiable to randomize clusters at the level of GP practices. Another limitation of the study is the delayed uptake of the intervention. We recommend researchers for future trials to incorporate transition period time for adaptation to the intervention during which clusters are not considered as either in the control or intervention condition [15]. We also doubt whether a RCT is the best instrument to evaluate such a complex intervention in such a complex setting, or whether implementation evaluation methods are better suited in such a case.

Qualitative research is necessary to further understand the impact of the intervention. Especially research on how perceived knowledge and skills of GPs are related to the use of an interdisciplinary consultation platform would be of interest as well as research on for which clinical questions such a platform is best suited.

We found positive trends in the same direction for both the PP and ITT analyses, with confidence intervals that largely overlap, despite the PP analysis not resulting in a significant difference between the intervention and control condition. This can be explained by the PP analysis having fewer observations in the intervention condition than the ITT analysis, resulting in lower statistical power. A possible explanation for the reduced estimate in the PP analyses may be that good performing clusters in terms of appropriate referrals were more likely not to take up the intervention. This was also reflected in the higher percentage of appropriate referrals in the control condition in the PP analysis compared to the ITT analysis (64.8% vs 62.9%). One could hypothesize is that for example older or more experiences GPs are less likely to take up new technology and are at the same time better referrers. These better performing clusters were subsequently included in the control condition reducing the effect of the intervention.

We observed a modest positive effect on the appropriate referral rate, but we are reluctant to make firm conclusions due to the complexity and dynamics within the study. In the future, platforms like these could serve as a support tool for use by GPs in daily practice, providing an opportunity to ask questions when in doubt about patient care or referral. As Dutch GPs have a crucial role in keeping specialist care accessible and affordable, this and similar platforms could lead to the avoidance of unnecessary patient visits to hospital.

## Supplementary Material

cmaf071_Supplementary_Data

## Data Availability

Information about the data and availability of the data can be found here: https://umcgresearchdatacatalogue.nl/UMCG/ssr-catalogue/all/collections/GPconsult.
