# Supplementary material for: Impact of an interdisciplinary digital consultation platform on general practitioner referrals for musculoskeletal symptoms: a stepped wedge cluster randomized trial
Source: Fam Pract. 2025 Sep 19;42(5):cmaf071. doi: 10.1093/fampra/cmaf071 (PMC12449199; doi:10.1093/fampra/cmaf071)
Supplement: cmaf071_Supplementary_Data [file cmaf071_supplementary_data.pdf]

Supplement 1.

Frequencies and percentages of total diagnosis codes for 4928 patients for knee, hip, shoulder and restgroup combined.

	Frequency	Percent
Knee	2445	49,6
Hip	1039	21,1
Shoulder	940	19,1
Miscellaneous	504	10,2
Total	4928	100,0

Patient characteristics of all referred patients for both conditions and total during the study period.

	Control	Intervention	Total
**Patients**	**1989**	**2939**	**4928**
Age (median)	60.8 (19-95)	60.9 (19-96)	60.0 (19-96)
Female (N (%))	1137 (57.2)	1608 (54.7)	2745 (55.7)
Appropriate referral (N (%))	1248 (62.7)	1977 (67.3)	3225 (65.4)
**Diagnosis**			
Shoulder (N (%))	392 (19.7)	548 (18.6)	940 (19.1)
Knee (N (%))	981 (49.3)	1464 (49.8)	2445 (49.6)
Hip (N (%))	415 (20.9)	624 (21.2)	1039 (21.1)
Other (N (%))	201 (10.1)	303 (10.3)	504 (10.2)
**Intervention**			
Repeat consultation (N (%))	724 (36.4)	1027 (34.9)	1751 (35.5)
Additional radiology (N (%))	589 (29.6)	906 (30.8)	1495 (30.3)
Intra-Articular injection (N (%))	136 (6.8)	224 (7.6)	360 (7.3)
Surgery (N (%))	209 (10.5)	384 (13.1)	593 (12.0)
